# The Reciprocal Risk Relationship of Dental Caries with NAFLD and Liver Fibrosis: Combined Evidence from the NHANES Study and Mendelian Randomization Analysis

**DOI:** 10.3290/j.ohpd.c_2714

**Published:** 2026-06-17

**Authors:** LiLi Xu, Xiaoyu Wang, Xiaofan Meng, Wenhui Wang, Honglin Liu, Hong Xiao, Chuanhua Zhang

**Affiliations:** a LiLi Xu Professor, Department of Clinical Laboratory, Zhongshan Second People’s Hospital, Zhongshan, China. Conceptualization, wrote original draft, read and agreed to the final version of the manuscript.; b Xiaoyu Wang MD Student, Division of Gastroenterology and Hepatology, Key Laboratory of Gastroenterology and Hepatology, Ministry of Health, Shanghai Institute of Digestive Disease, Renji Hospital, School of Medicine, Shanghai Jiao Tong University, Shanghai, China. Conceptualization, methodology, formal analysis, wrote original draft, read and agreed to the final version of the manuscript.; c Xiaofan Meng Lecturer, Shanghai JingAn District ZhaBei Central Hospital, Shanghai, China. Conceptualization, methodology, investigation, formal analysis, read and agreed to the final version of the manuscript.; d Wenhui Wang Dentist, School of Stomatology, Nanjing Medical University, Nanjing, China. Methodology, formal analysis, wrote original draft, read and agreed to the final version of the manuscript.; e Honglin Liu PhD Student, Department of Clinical Laboratory, Zhongshan Second People’s Hospital, Zhongshan, China; Department of Clinical Laboratory, The Third People’s Hospital of Liupanshui, Liupanshui, Guizhou, China. Conceptualization, supervision, read and agreed to the final version of the manuscript.; f Hong Xiao Lecturer, Department of Clinical Laboratory, The Third People’s Hospital of Liupanshui, Liupanshui, Guizhou, China. Visualization, read and agreed to the final version of the manuscript.; g Chuanhua Zhang Professor, Department of Clinical Laboratory, The Third People’s Hospital of Liupanshui, Liupanshui, Guizhou, China. Investigation, visualization, supervision, funding acquisition, read and agreed to the final version of the manuscript. The first five authors contributed equally to this manuscript.

**Keywords:** dental caries, liver fibrosis/cirrhosis, Mendelian randomization, National Health and Nutrition Examination Survey, non-alcoholic fatty liver disease.

## Abstract

**Purpose:**

To explore the relationship between dental caries and non-alcoholic fatty liver disease (NAFLD) and its advanced stage of liver fibrosis, and to confirm causality by employing a bidirectional two-sample Mendelian Randomization (MR) analysis to confirm causality.

**Materials and Methods:**

6650 participants of the National Health and Nutrition Examination Survey (NHANES), 2017–2020 were included. Two multivariable logistic regression models were used to evaluate the relationship between untreated dental caries (UDC) and liver conditions of NAFLD and fibrosis, with adjustments for demographics, lifestyle, and medical history. Furthermore, two-sample MR was performed with caries as exposure and NAFLD with NAFLD-related conditions as outcome, and vice versa for bidirectional causality validation.

**Results:**

In observational research, UDC was notably associated with NAFLD (OR [odds ratio]: 1.40, 95% CI [confidence interval]: 1.06–1.86) and significant fibrosis (SF) (OR: 1.29, 95% CI: 1.03–1.62). NAFLD and SF were statistically significantly associated with UDC (OR: 1.40, 95% CI: 1.08–1.83; OR: 1.62, 95% CI: 1.26–2.08). In the MR analysis, caries posed a statistically non-significant risk for liver conditions. In contrast, liver conditions non-significantly protected against caries (NAFLD: OR: 0.99, 95% CI: 0.98−1.01; fibrosis: OR: 0.99, 95% CI: 0.99−1.00; cirrhosis: OR: 0.99, 95% CI: 0.99−1.00; fibrosis/cirrhosis: OR: 1.00, 95% CI: 0.98−1.02).

**Conclusion:**

Observational studies suggested a statistically significant association between UDC and liver conditions of NAFLD and fibrosis. However, MR suggested a statistically non-significant causal relationship between caries and liver conditions; in contrast, liver conditions had a non-significant protective effect on caries.

Dental caries, commonly known as tooth decay, refers to the chronic, bacterial, and progressive destruction of dental hard tissue, including the tooth enamel and underlying dentin. *Streptococcus mutans* (*S. mutans*) is a major cariogenic pathogen.^33^ According to an analysis of the Global Burden of Disease 2017 Study focusing on oral conditions, the age-standardized prevalence of untreated caries in deciduous and permanent teeth was 7.8% (95% uncertainty interval [UI], 6.5% to 9.1%) and 29.4% (95% UI, 26.8% to 32.2%), respectively. Although easy to prevent by developing healthy oral hygiene habits and reducing sugar consumption, its leading prevalence worldwide among young and old did not seem to decline statistically significantly, as the percentage change in the number of prevalent cases between 1990 and 2017 was -7.9% and -8.8%, respectively.^19^ The high prevalence of caries is to some extent attributed to the modern lifestyle, including high-sugar, high-fat diets, circadian rhythm disruption, and sedentary behavior, which help form a favorable environment for oral bacteria and weaken the regulation of the inflammatory immune system, resulting in the acceleration of caries.^26,28^ Caries is also a catalyst of systemic diseases: eating dysfunction leads to under-mastication and indigestion, while pain and sensitivity can lead to a decreased willingness to perform oral hygiene measures. Over time, malnutrition and immune dysfunction could result in a higher likelihood of metabolic syndrome, including diabetes and non-alcoholic fatty liver disease (NAFLD, now named metabolic associated fatty liver disease, MAFLD or metabolic dysfunction-associated steatotic liver disease, MASLD), as well as bacterial flora disturbances in the alimentary tract.^40^ NAFLD is a metabolic disorder marked by the accumulation of extra fat in the liver, despite a lack of the common causal factors, e.g., excessive alcohol consumption, immune disorders, etc.^34^ Both natural and environmental factors, including insulin resistance, genetic variations, obesity, sedentary lifestyle, and unhealthy dietary habits as mentioned above can contribute to NAFLD,^42,56^ leading to its rapidly increasing prevalence up to approximately 25% of the global population in the 21st century.^54^ With an in-depth investigation of the role of microbiota in obesity-related metabolic disorders, *S. mutans* has also been found to aggravate NAFLD and fibrosis in non-alcoholic steatohepatitis (NASH), a condition in the spectrum of NAFLD.^29,48,49^ Liver fibrosis and cirrhosis are both progressive stages of common liver conditions, and NAFLD is the third most rapidly emerging major cause of fibrosis.^20^ If left untreated, it advances to an irreversible process of cirrhosis, which often necessitates liver transplantation.^7^ Progression to liver fibrosis/cirrhosis can result in complications, including liver failure and hepatocellular carcinoma (HCC), as well as systematic complications such as cardiovascular disease and diabetes.^17,23,37^

Current research primarily involves observational studies that highlight the interrelated risks between dental caries and liver conditions.^4,14,52^ Owing to the lack of further investigation on mechanical validation, the causal relationship between caries and NAFLD remains unclear. One hypothesis suggests that caries may influence obesity through shared risk factors including diet, biological indicators such as reduced saliva production and distinct oral microbial profiles,^3,13^ and lifestyle. Recent studies have advocated the “multiple crosstalk” hypothesis, stressing that dysregulation of other organs as well as the liver in obesity could aggravate obesity-related hepatic steatosis, ultimately progressing to obesity-associated NAFLD. Mendelian randomization (MR) is an emerging approach in which genetic variants are used as instrumental variables (IVs) to deduce causality between exposure and outcome. Including data from state-of-the-art liver ultrasound transient elastography used for the first time by NHANES 2017-2020, our study aimed to explore the relationship between caries and NAFLD and its advanced stage of liver fibrosis. Furthermore, a bidirectional two-sample MR analysis was employed to confirm causality, making important contributions to our understanding of the mutual risks of caries for NAFLD and its advanced conditions of liver fibrosis, cirrhosis, and fibrosis/cirrhosis.

## MATERIALS AND METHODS

**Fig 1:**
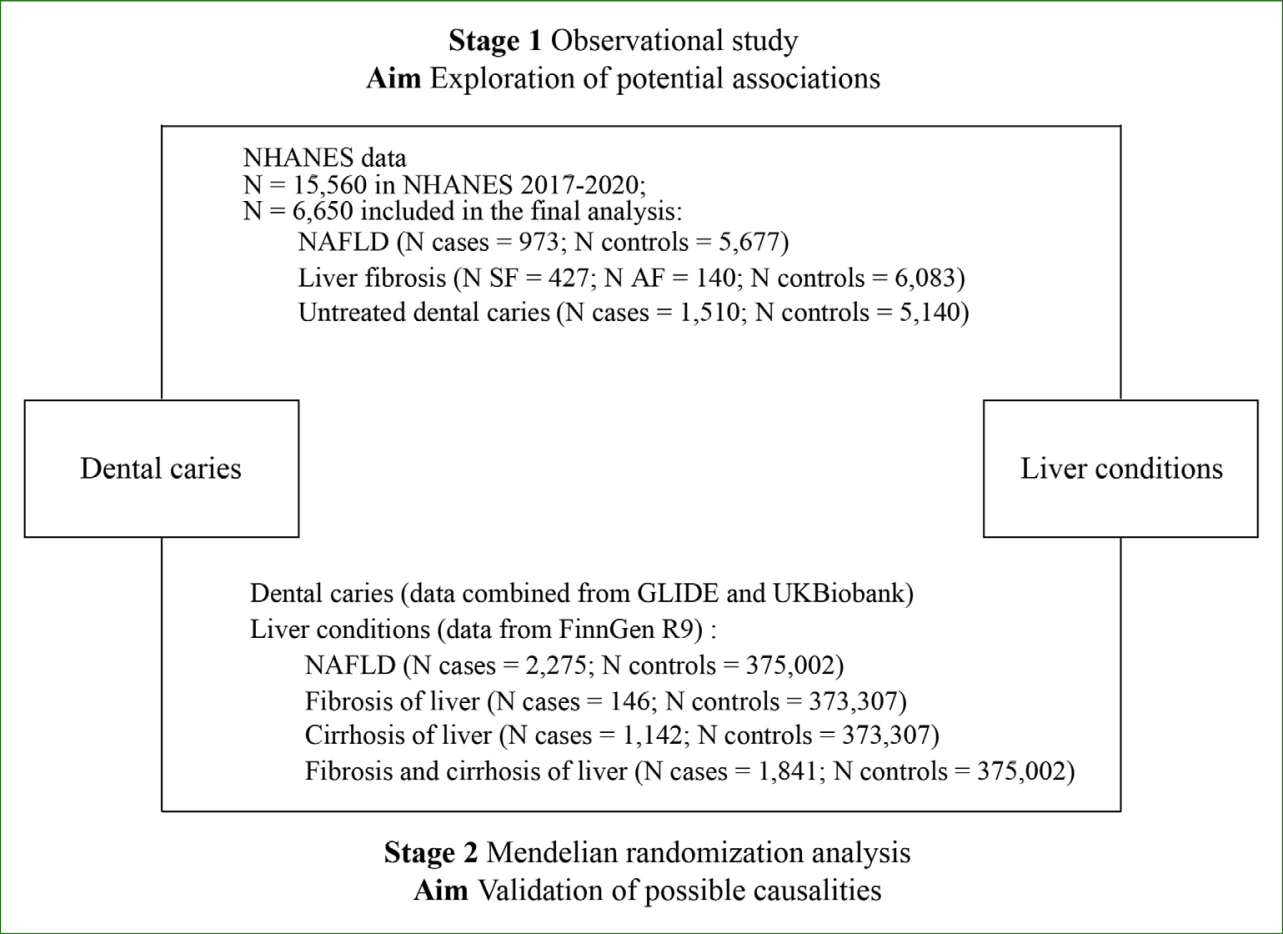
Overall study design. NAFLD, non-alcoholic fatty liver disease; SF, significant fibrosis; AF, advanced fibrosis.

This study included two stages, as shown in Fig 1. In stage 1, multivariable regression analysis was performed using data extracted from the National Health and Nutrition Examination Survey (NHANES) database to determine the association between caries and liver conditions. In stage 2, MR analysis employed summary statistics from a GWAS (Genome-Wide Association Studies) study and FinnGen to validate the bidirectional causality.

### NHANES

#### Data sources and study population 

**Fig 2:**
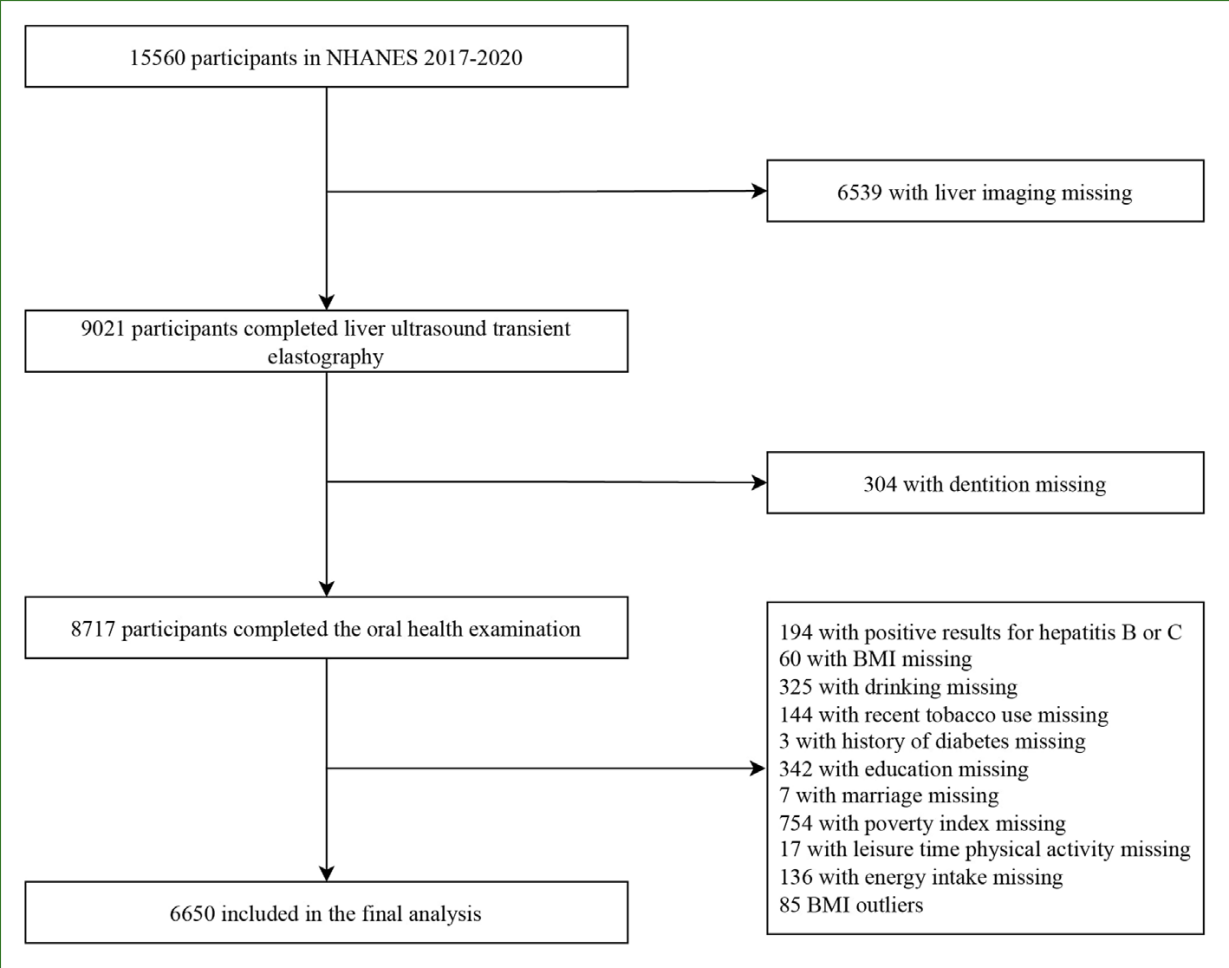
Data analysis screening flowchart in NHANES.

NHANES comprises a series of consecutive cross-sectional surveys focused on the United States population. It generates a nationally representative sample method utilizing a multi-stage probability sampling procedure and assesses health and nutritional status. The survey included subjective data from interviews conducted in participants’ households and objective data from physical evaluations. All participants provided written informed consent, and the National Center for Health Statistics Ethics Review Board approved the study. After excluding participants with viral hepatitis, extreme body mass index (BMI), and those without liver stiffness measurement (LSM), dentition examination, or other significant covariates, 6650 participants were identified in this study (Fig 2).

#### Assessment of caries and liver conditions

Caries was diagnosed during the oral health examination. Untreated dental caries (UDC) was defined exclusively as untreated coronal decay, excluding treated teeth and those extracted due to caries. NAFLD and stage of liver fibrosis were evaluated using LSM, hepatic fat quantified as the Controlled Attenuation Parameter (CAP), and the interquartile range divided by the median can be used to assess the reliability of liver stiffness measurements.^12^ All data were available through Vibration-Controlled Transient Elastography (VCTE). A CAP median score ≥285 dB/m was used to diagnose NAFLD. LSMs of 8~13.1 kPa and ≥13.1 kPa, respectively, were used to identify significant fibrosis (SF) and advanced fibrosis (AF).

#### Assessment of covariates

The assessment of covariates is critically important in research as it aids in controlling potential confounding factors, ensuring a more accurate and reliable evaluation of the primary relationships. In our study, we selected a range of covariates to gain a better understanding of the relationship between caries and liver conditions. Covariates including sex, race, age, alcohol consumption, recent tobacco use, history of diabetes, education, marital status, family monthly poverty level index, energy intake, and leisure time physical activity (LTPA) were collected through standardized questionnaires. BMI was calculated as weight (kg) divided by height squared (m^2^) with the original data of each participant obtained from physical examinations.

#### Statistical analyses

All analyses were performed using SPSS (version 26) and R (version 4.3.2). NHANES 2017-2020 data were incorporated into all our analyses. Four-year Mobile Examination Center examination weights were employed to adjust for non-response and unequal selection probabilities inherent to the complex multistage probability sampling design according to the NHANES Analytic and Reporting Guidelines. To assess variances in continuous and categorical variables between the NAFLD vs non-NAFLD groups, different stages of LSM, or UDC vs no UDC, we employed the chi-squared test for categorical variables and ANOVA for continuous variables. Multivariable logistic regression was performed to investigate the effects of caries on the likelihood of NAFLD and LSM elevation, and vice versa. Covariates were selected based on both theoretical relevance and clinical significance. Potential multicollinearity among covariates (e.g., BMI and energy intake) was assessed using the Variance Inflation Factor (VIF). All variables yielded a VIF <5, indicating no severe multicollinearity. Model 1 was only adjusted for race, sex, and age, and Model 2 was adjusted for education level, marital status, race, sex, age, recent smoking, LTPA, BMI, family monthly poverty level index, energy intake, and history of diabetes. Additionally, to evaluate whether the association between UDC and liver conditions differed by sex and age, formal statistical tests for interaction were conducted by incorporating cross-product terms in the regression models. Statistical significance was set at 0.05. Odds ratios (OR), matching 95% confidence intervals (CIs), and the resulting two-sided significance levels were computed. Furthermore, to determine the quantitative effect of caries severity on NAFLD and SF, we added NHANES analyses based on classification of the number of untreated carious lesions: (1) zero; (2) one to two; or (3) more than two, instead of simple binary classification by (1) with or (2) without caries.

### MR Analyses

#### Study design description

Eight two-sample MR analyses were performed to verify the bidirectional association between caries and liver conditions. The forward MR analyses considered caries as the exposure and liver conditions including NAFLD, fibrosis, cirrhosis, and fibrosis/cirrhosis as the outcome; in the reverse MR analyses, liver conditions, as the exposure and caries as the outcome. As the study was based on publicly accessible summary statistics, no ethical approval was required. The MR analyses were performed using R (version 4.3.2), and notably, three packages were employed during the data process and visualization: “TwoSampleMR”,^22^ “MRPRESSO”,^50^ and “forestploter”.^15^ The study was conducted following the STROBE-MR (Strengthening the Reporting of Observational Studies in Epidemiology using Mendelian Randomization) checklist.^45^

#### Data sources and instrumental variables selection for MR analyses

Summary statistics of caries (n: 487,823) represented by the results of the z-score meta-analysis of decayed, missing, and ﬁlled tooth surfaces (DMFS) and dentures were obtained from a GWAS study published in 2019, in which DMFS data were obtained from the GeneLifestyle Interactions in Dental Endpoints (GLIDE) consortium and data of dentures were obtained from the UK Biobank (UKB).^21^ As a combination of clinical and self-reported traits, the GWAS dataset of DMFS/dentures revealed the progression of caries. The diseases were diagnosed by an experienced assessor or a dental professional as described in the original GWAS study.^44^ The data applied in our study were taken from meta-analyses of multi-regional studies comprising individuals of genetically confirmed European descent, including mixed populations such as the Hispanic Community Health Study/Study of Latinos (HCHS/SOL). Data of liver conditions including traits of NAFLD (n: 2,275 cases/375,002 controls), fibrosis (n: 146 cases/373,307 controls), cirrhosis (n:1,142 cases/373,307 controls), and fibrosis/cirrhosis (n: 1,841 cases/366,450 controls) were obtained from the FinnGen database, which originated from a large public project launched in Finland, covering over 370,000 Finnish biobank participants.^25^ The diseases were diagnosed according to ICD-10. Detailed data of GWAS studies included in the MR analyses are shown in Table A1 (see Appendix: https://www.quintessence-publishing.com/quintessenz/journals/articles/downloads/ohpd_2026_8360_xu_supplements.pdf).

**Fig 3:**
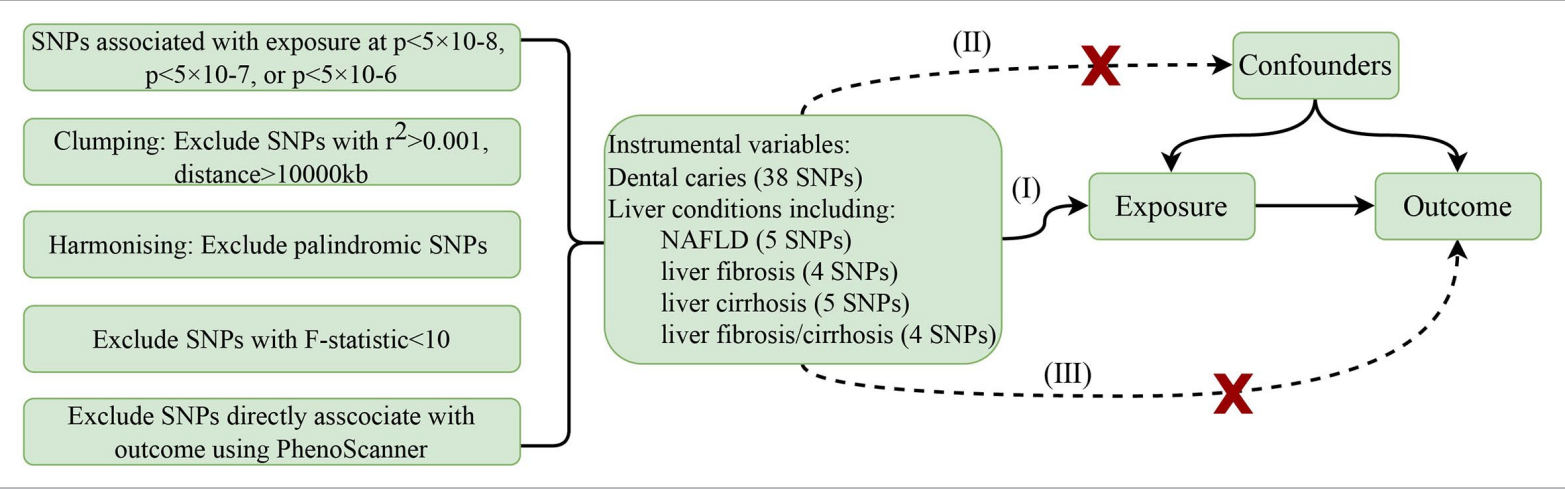
The screening of instrumental variables and the three crucial IV assumptions in MR analysis. Assumption Ⅰ: IVs (instrumental variables) should be strongly linked to the exposure. Assumption Ⅱ: IVs should not be influenced by any confounders. Assumption Ⅲ: IVs applied should only act on the outcome through the exposure instead of any other pathway. SNP: single nucleotide polymorphism; NAFLD: non-alcoholic fatty liver disease.

Figure 3 illustrates the screening of the IVs used in our study. Single nucleotide polymorphism (SNPs), the IVs used in the analysis, were first chosen at a genome-wide significance threshold. As too few SNPs would lead to unstable estimates,^10^ the threshold of SNPs was set at p<5×10^-8^ (caries), p<5×10^-7^ (NAFLD, cirrhosis, fibrosis/cirrhosis) and p<5×10^-6^ (fibrosis). Then, SNPs located in the target gene region and its neighboring >10,000 kb window were extracted, and linkage disequilibrium (LD) clumping (r^2^<0.001) in the European 1000 Genome datasets was applied. Palindromic SNPs with intermediate allele frequencies were eliminated when harmonizing the exposure and outcome data (action = 3). F-statistics were calculated and SNPs with F<10 were excluded. The PhenoScanner database was used to minimize the interference of confounders related to SNPs with p <1 ×10^-5^ and to ensure the reliability of results.^24^ Table A2 lists detailed information on the SNPs used as IVs in this study. For caries, 38 SNPs were used for analysis; for liver conditions including NAFLD, liver fibrosis, liver cirrhosis, and liver fibrosis/cirrhosis, 5, 4, 5 and 4 SNPs were used for analysis.

#### Statistical analysis

The inverse variance weighted (IVW) method is the principal statistical method used to estimate potential bidirectional causality between caries and liver conditions. Regarding supplementary methods, we added the MR Egger, weighted median, and weighted mode for validation. The IVW method assumes the premise that all the core assumptions of MR hold, and the heterogeneities were calculated using Cochran’s Q statistic. Sensitivity analyses were performed to resolve the pleiotropic effects in the causal estimates, as pleiotropic instrumental variables may have introduced bias into the results. In this instance, the MR-Egger regression was applied, allowing us to precisely estimate the causal relationship.^8,9^ The intercept term of the MR-Egger regression indicates the average pleiotropic effect of the IVs. The symmetry of funnel plots also suggested the extinction of horizontal pleiotropy. Leave-one-out analyses were performed to assess the consistency and reliability of the results. Additionally, the MR Pleiotropy Residual Sum and Outlier (MR-PRESSO) test was conducted to assess the existence of pleiotropy. Local association plots were created using LocusZoom.^35^

## RESULTS

### Baseline Characteristics of Participants with NAFLD, SF, AF and UDC in NHANES

Participants with NAFLD or elevated LSM tended to be over 50 years of age, and the percentage of male patients was approximately 60%. In contrast to participants without NAFLD or elevated LSM, those with NAFLD or elevated LSM had a higher prevalence of UDC, more elevated CAP and LSM, metabolic disorders including diabetes and obesity, history of recent tobacco use, and absence of leisure-time physical activity. Participants with UDC were more likely to be older, male, and current smokers; have higher CAP, LSM, and BMI; poorer poverty index and spend less leisure time doing physical activity; and with recent tobacco use and history of diabetes. Compared to healthy controls, those with UDC tended to have higher energy intake levels (Table 1).

**Table 1:** Baseline characteristics of study population in NHANES 2017–2020

Characteristics	Total (N = 6,650)	NAFLD			Liver fibrosis				UDC		
		Yes (N = 973)	No (N = 5,677)	p-value	Normal (N = 6,083)	SF (N = 427)	AF (N = 140)	p-value	0 (N = 5,140)	>0 (N = 1,510)	p-value
Age, years, Mean (SE)	44.6 (0.3)	52.4 (0.6)	43.2 (0.3)	<0.01	43.7 (0.3)	52.1 (0.9)	57.1 (1.5)	<0.01	44.1 (0.3)	46.0 (0.5)	0.01
**Sex, n (%)**				<0.01				<0.01			<0.01
Male	3318 (49.9)	606 (62.3)	2712 (47.8)		2978 (49.0)	256 (60.0)	84 (60.0)		2503 (48.7)	815 (54.0)	
Female	3332 (50.1)	367 (37.7)	2965 (52.2)		3105 (51.0)	171 (40.0)	56 (40.0)		2637 (51.3)	695 (46.0)	
UDC, Mean (SE)	0.7 (0.03)	0.8 (0.1)	0.7 (0.03)	0.18	0.7 (0.03)	0.9 (0.1)	0.9 (0.2)	0.05	0	3.2 (0.1)	<0.01
CAP, dB/m, Mean (SE)	258.0 (0.8)	329.7 (1.0)	245.7 (0.8)	<0.01	253.5 (0.8)	303.7 (3.1)	314.2 (5.9)	<0.01	256.5 (0.9)	263.0 (1.7)	0.01
LSM, kPa, Mean (SE)	5.6 (0.05)	6.7 (0.2)	5.4 (0.1)	<0.01	4.9 (0.02)	9.7 (0.1)	24.2 (1.4)	<0.01	5.5 (0.1)	5.9 (0.1)	<0.01
Body mass index, kg/m^2^ Mean (SE)	28.7 (0.1)	32.6 (0.2)	28.1 (0.1)	<0.01	28.2 (0.1)	33.9 (0.4)	34.7 (0.7)	<0.01	28.4 (0.1)	29.7 (0.2)	<0.01
**Ethnicity, n (%)**				<0.01				0.03			<0.01
Mexican American	806 (12.1)	135 (13.9)	671 (11.8)		727 (12.0)	57 (13.4)	22 (15.7)		650 (12.6)	156 (10.3)	
Other Hispanic	644 (9.7)	81 (8.3)	563 (9.9)		590 (9.7)	41 (9.6)	13 (9.3)		502 (9.8)	142 (9.4)	
Non-Hispanic White	2449 (36.8)	396 (40.7)	2053 (36.2)		2226 (36.6)	158 (37.0)	65 (46.5)		1985 (38.6)	464 (30.7)	
Non-Hispanic Black	1666 (25.1)	187 (19.2)	1479 (26.1)		1524 (25.0)	118 (27.6)	24 (17.1)		1105 (21.5)	561 (37.2)	
Other Race – Including Multi-Racial	1085 (16.3)	174 (17.9)	911 (16.0)		1016 (16.7)	53 (12.4)	16 (11.4)		898 (17.5)	187 (12.4)	
**Educational levels, n (%)**				0.91				0.02			0.08
Less than high school	1951 (29.3)	287 (29.5)	1664 (29.3)		1812 (29.8)	109 (25.5)	30 (21.4)		1535 (29.9)	416 (27.5)	
High school or above	4699 (70.7)	686 (70.5)	4013 (70.7)		4271 (70.2)	318 (74.5)	110 (78.6)		3605 (70.1)	1094 (72.5)	
**Marriage status, n (%)**				<0.01				<0.01			0.29
Have a partner or is married	3330 (50.1)	562 (57.8)	2768 (48.8)		3010 (49.5)	232 (54.3)	88 (62.9)		2592 (50.4)	738 (48.9)	
Single or widowed	3320 (49.9)	411 (42.2)	2909 (51.2)		3073 (50.5)	195 (45.7)	52 (37.1)		2548 (49.6)	772 (51.1)	
**Poverty status, n (%)**				0.02				0.42			<0.01
≤1.30	2110 (31.7)	346 (35.6)	1764 (31.1)		1928 (31.7)	139 (32.6)	43 (30.7)		1431 (27.8)	679 (45.0)	
1.30-3.50	983 (14.8)	141 (14.5)	842 (14.8)		901 (14.8)	68 (15.9)	14 (10.0)		708 (13.8)	275 (18.2)	
>3.50	3557 (53.5)	486 (49.9)	3071 (54.1)		3254 (53.5)	220 (51.5)	83 (59.3)		3001 (58.4)	556 (36.8)	
**Energy intake levels, n (%)**				0.12				0.28			<0.01
Inadequate	2808 (42.2)	435 (44.7)	2373 (41.8)		2562 (42.2)	177 (41.4)	69 (49.3)		2175 (42.3)	633 (41.9)	
Adequate	2693 (40.5)	389 (40.0)	2304 (40.6)		2472 (40.6)	177 (41.5)	44 (31.4)		2121 (41.3)	572 (37.9)	
Excessive	1149 (17.3)	149 (15.3)	1000 (17.6)		1049 (17.2)	73 (17.1)	27 (19.3)		844 (16.4)	305 (20.2)	
**Recent tobacco use, n (%)**				<0.01				0.47			<0.01
Yes	1240 (18.6)	110 (11.3)	1130 (19.9)		1143 (18.8)	76 (17.8)	21 (15.0)		749 (14.6)	491 (32.5)	
No	5410 (81.4)	863 (88.7)	4547 (80.1)		4940 (81.2)	351 (82.2)	119 (85.0)		4391 (85.4)	1019 (67.5)	
**Leisure time physical activity, minutes, n (%)**				<0.01				<0.01			<0.01
0	2955 (44.5)	547 (56.2)	2408 (42.4)		2624 (43.2)	238 (55.7)	93 (66.5)		2118 (41.2)	837 (55.4)	
0-150	1080 (16.2)	152 (15.6)	928 (16.4)		987 (16.2)	76 (17.8)	17 (12.1)		878 (17.1)	202 (13.4)	
≥150	2615 (39.3)	274 (28.2)	2341 (41.2)		2472 (40.6)	113 (26.5)	30 (21.4)		2144 (41.7)	471 (31.2)	
**History of diabetes, n (%)**				<0.01				<0.01			<0.01
Yes	805 (12.1)	270 (27.7)	535 (9.4)		611 (10.0)	138 (32.3)	56 (40.0)		575 (11.2)	230 (15.2)	
No	5845 (87.9)	703 (72.3)	5142 (90.6)		5472 (90.0)	289 (67.7)	84 (60.0)		4565 (88.8)	1280 (84.8)	
NAFLD: non-alcoholic fatty liver disease; SF: significant fibrosis; AF: advanced fibrosis; SE: standard error of mean; UDC: untreated dental caries; CAP: controlled attenuation parameter, a measure of liver steatosis; LSM: liver stiffness measurement.											

### Observational Association of UDC with Liver Conditions in NHANES

#### The effect of UDC on liver conditions in NHANES

**Fig 4:**
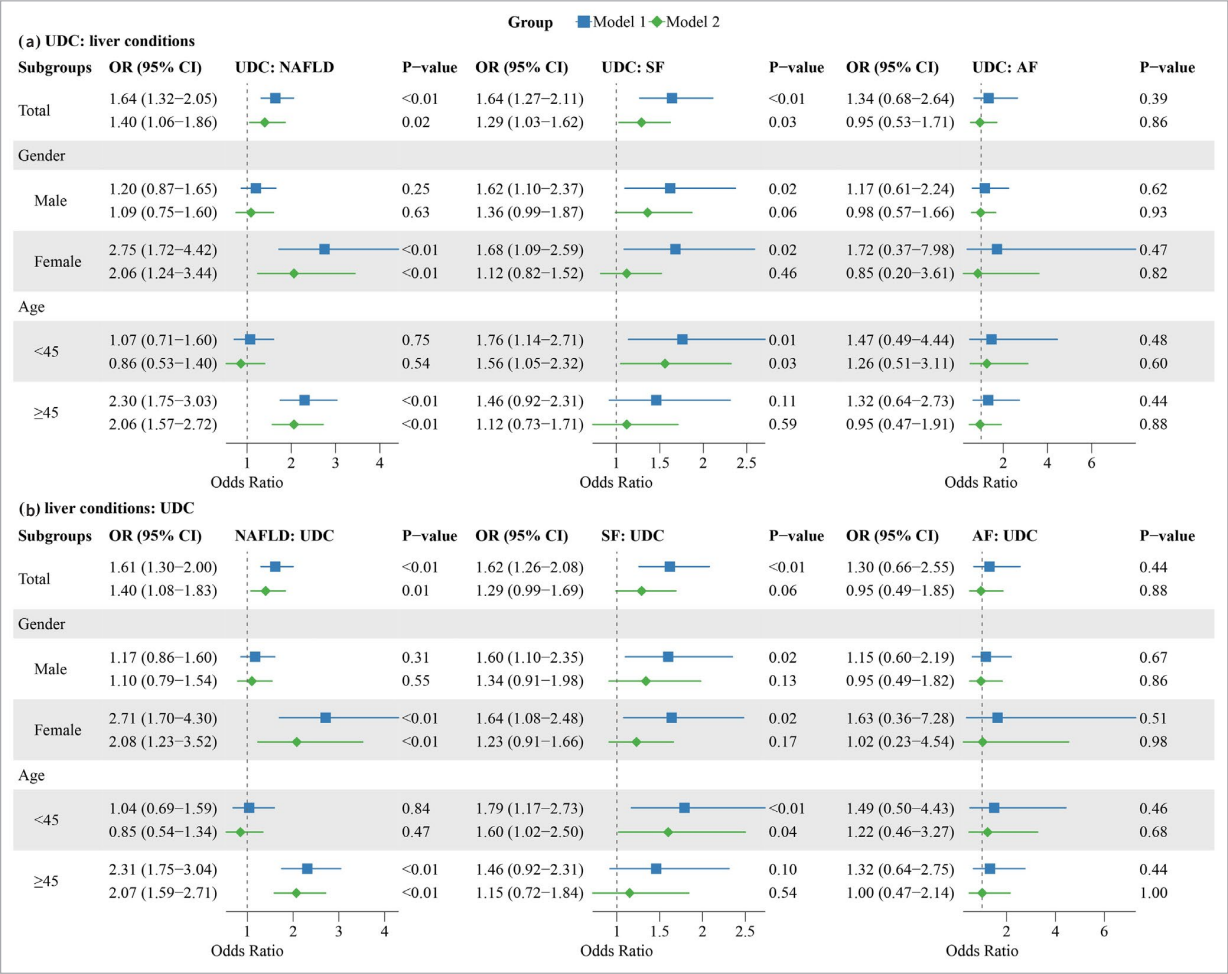
The bidirectional association between dental caries and liver conditions according to NHANES. (A) The effect of UDC on the prevalence of NAFLD, SF and AF, and (B) the effect of NAFLD, SF and AF on the prevalence of UDC, with data subgroups divided in terms of total population, sex and age, each analyzed with two models. Model 1 was only adjusted for race, sex and age, and Model 2 was adjusted for education level, marriage, race, sex, age, recent smoking, LTPA, BMI, family monthly poverty level index, energy intake and history of diabetes. UDC: untreated dental caries; NAFLD: non-alcoholic fatty liver disease; SF: significant fibrosis; AF: advanced fibrosis; OR: odds ratio; CI: confidence interval.

The ORs, 95% CIs, and p-values for all cases, as well as subgroups of sex and age in the two covariate-adjusted regression models, are visualized as forest plots (Fig 4a). Among the participants, a higher total potential of NAFLD was statistically significantly associated with UDC (OR: 1.40, 95% CI: 1.06–1.86). The risk effect of UDC displayed a sex and age orientation, as it increased in women (OR: 2.06, 95% CI: 1.24–3.44) and in those over 45 years old (OR: 2.06, 95% CI: 1.57–2.72).

The effect of UDC on SF was also statistically significant (OR: 1.29, 95% CI: 1.03–1.62), despite a statistically significant but different direction of sex and age orientation, according to the potential in males (OR: 1.62, 95% CI: 1.75–3.03) and in those under 45 years old (OR: 1.56, 95% CI: 1.05–2.32). However, no statistically significant effect of UDC could be found for AF, because the results of the two models contradicted each other, except for the risk tendency of UDC in those under 45 years old (OR: 1.26, 95% CI: 0.51–3.11). In addition, the results of the three-category variable analysis further verified our previous conclusion that caries had a harmful influence on the liver, which was reinforced with early-stage dental damage (Fig A1: https://www.quintessence-publishing.com/quintessenz/journals/articles/downloads/ohpd_2026_8360_xu_supplements.pdf).

#### The effect of liver conditions on UDC in NHANES

We also explored the probable association between pathological hepatic changes and caries (Fig 4b). NAFLD showed a statistically significant overall risk effect (OR: 1.40, 95% CI: 1.08–1.83), and so did SF (OR: 1.62, 95% CI: 1.26–2.08), but no statistically significant or exact tendency in AF. In the sex and age subgroups, NAFLD was more common in females (OR: 2.08, 95% CI: 1.23–3.52) and seniors (OR: 2.31, 95% CI: 1.75–3.04, and OR: 2.07, 95% CI: 1.59–2.71). In contrast, SF tended to be present among younger individuals (OR: 1.79, 95% CI: 1.17–2.73, and OR: 1.60, 95% CI: 1.02–2.50).

Using the latest LSM data from NHANES 2017–2020, we were able to estimate the effect of pathological liver-stiffness changes on caries more accurately. Additional NHANES analyses were conducted in the categories of SF and AF defined by LSM, instead of the binary categorization with or without the liver conditions. The influence of SF significantly supported the conclusion that moderate liver damage boosted caries incidence, especially in male and younger individuals (Fig A2).

### Causal Relationship Between Caries and Liver Conditions in MR

**Fig 5:**
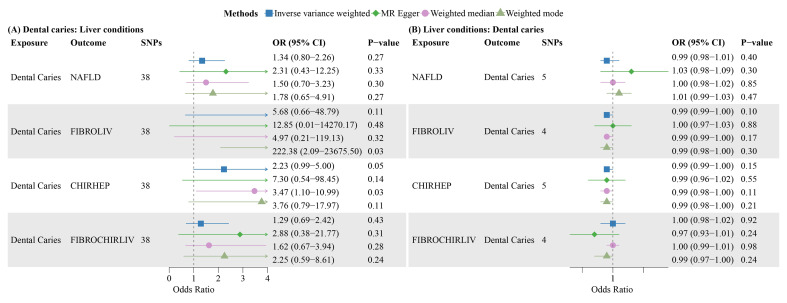
Causal relationship between dental caries and liver conditions in MR analysis. The causal effect of (a) dental caries on liver conditions and (b) liver conditions on dental caries. SNP: single nucleotide polymorphism; OR: odds ratio; CI: confidence interval; NAFLD: non-alcoholic fatty liver disease; FIBROLIV: liver fibrosis; CHIRHEP: liver cirrhosis; FIBROCHIRLIV: fibrosis and cirrhosis of liver.

In the MR analysis, in which caries served as an exposure, 38 SNPs were used after selection. The IVW results implied an overall statistically non-significant association between genetically predicted caries and an elevated risk of liver conditions (NAFLD: OR: 1.34, 95% CI: 0.80−2.26; fibrosis: OR: 5.68, 95% CI: 0.66−48.79; cirrhosis: OR: 2.23, 95% CI: 0.99−5.00; fibrosis/cirrhosis: OR: 1.29, 95% CI: 0.69−2.42). Supplementary methods showed consistent coherent trends, especially statistically significant connections were shown for the effect of caries on cirrhosis in weighted median (OR: 3.47, 95% CI: 1.10−10.99) (Fig 5a). Heterogeneity analysis revealed no statistically significant potential heterogeneity in the effect of caries on liver conditions (NAFLD: IVW P: 0.88; fibrosis: IVW P: 0.30; cirrhosis: IVW P: 0.17; fibrosis/cirrhosis: IVW P: 0.21). The Egger intercept showed no horizontal pleiotropy between the effect of caries and liver conditions (Egger intercept P: NAFLD:0.51; fibrosis: 0.81; cirrhosis: 0.35; fibrosis/cirrhosis: 0.42) (Tables A3 and A4).

We also explored the causal effect of liver conditions on caries using reverse MR analysis, in which 5, 4, 5, and 4 SNPs screened for NAFLD, liver fibrosis, liver cirrhosis, and liver fibrosis/cirrhosis, respectively. According to IVW results, genetically predicted liver conditions acted as statistically non-significant protective factors to caries (NAFLD: OR: 0.99, 95% CI: 0.98−1.01; fibrosis: OR: 0.99, 95% CI: 0.99−1.00; cirrhosis: OR: 0.99, 95% CI: 0.99−1.00; fibrosis/cirrhosis: OR: 1.00, 95% CI: 0.98−1.02). Additionally, the supplementary methods of MR Egger, weighted median, and weighted mode exhibited a statistically non-significant and inconsistent causal connection with the effect of liver conditions on caries (Fig 5b). The subsequent heterogeneity analysis showed no statistically significant potential heterogeneity in the effect of liver conditions on caries (NAFLD: IVW P: 0.31; fibrosis: IVW P: 0.89; cirrhosis: IVW P: 0.90; fibrosis/cirrhosis: IVW P: 0.08). The Egger intercept showed no horizontal pleiotropy in terms of the effect of liver conditions on caries (Egger intercept P: NAFLD:0.21; fibrosis: 0.87; cirrhosis: 0.79; fibrosis/cirrhosis: 0.21) (Tables A3 and A4).

Figures A3 and A4 illustrate the MR sensitivity analysis results in scatter plots, leave-one-out analysis, and funnel plots. Figure A5 depicts the regional association plots for each trait including the lead SNP and nearest genes.

## DISCUSSION

To the best of our knowledge, this study is the first to examine the association between caries and liver conditions using bidirectional analyses of the NHANES and large-scale genetic data. The findings underscore the need for healthcare providers to consider oral health status when assessing a patient’s overall health and to apply a more holistic approach to healthcare. There is also a need to raise public awareness of the connection between oral health and systemic diseases. Evidence from observational studies in the U.S. population suggests that UDC is significantly associated with an elevated occurrence of NAFLD, and conversely, NAFLD has a higher potential for UDC in the American population. MR analyses showed a similar tendency but no statistically significant causal relationship in the observed European population. In addition, we investigated the relationship between caries and liver conditions, including fibrosis, cirrhosis, and fibrosis/cirrhosis which has not yet been explored before. Despite the statistically significant positive association of UDC with LSM, a credible result indicating liver condition severity, MR analyses only yielded a similar tendency without statistical significance. Our results highlight the need for further research to elucidate the underlying mechanisms and to determine whether there is a direct causal link or whether other confounding factors are at play. The robustness of the evidence for every causation was verified by the sensitivity analyses. As a randomized controlled trial, MR is greatly affected by randomness, which may result in statistically significant differences between experimental and control groups. Thus, there are a few unstable estimated effects with wide CIs, presenting as extremely high values and wide CIs in the Figures.

Consumption of highly processed products can lead to increased sucrose intake, a nutrient that mediates the formation and adhesion of cariogenic *S. mutans* biofilms.^31,38,51^ Bacteria from carious lesions enter the bloodstream and spread immune responses and inflammation to organs all over the body, leading to systematic diseases.^32,43^ An MR analysis conducted in 2023 found various modifiable risk factors genetically associated with NAFLD, including alcohol frequency, serum parameters such as gamma-glutamyl transferase (GGT) and aspartate transaminase (AST), and health conditions such as fat mass of limbs and trunk, and type 2 diabetes.^53^ Metabolic syndrome –including insulin resistance, hyperglycemia, and dyslipidemia – were found to be associated with carious teeth.^47^ Based on a Brazilian population-based cohort, researchers found that the insulin resistance phenotype (defined with blood lipid indicators) was associated with the chronic oral disease burden (latent variable including caries).^27^ However, there have only been a few studies of the association between caries and liver conditions. Using data from NHANES, 1988 to 1994, a previous study discussed the connection between NAFLD and several oral disorders including caries, in which NAFLD was defined using ultrasonography, fibrosis score, fatty liver index, and US fatty liver index. Som evidence pointed to an association between NAFLD and UDC, but this was not statistically significant in terms of overall caries experience.^52^ According to our observational analysis using data from NHANES 2017-2020, UDC has an influence on the potential development of NAFLD and liver fibrosis, and the quantified newly-available imaging data of liver condition further supported this observation. However, few studies highlighted the effect of liver conditions on oral disease. Although liver transplantation (LTx), the treatment of end-stage liver disease, was found to be associated with an increased risk of caries in both pre-LTx and post-LTx populations, patients at the pre-LTx stage showed no statistically significant increase in the total number of UDC.^30^ Together with the increasing knowledge of microbiota, the theory of the oral-gut-liver axis has emerged. Its imbalance is speculated to trigger liver inflammation and dysregulated immune response, although most evidence is based on periodontal diseases. Many oral microorganisms, including *Streptococcus*, have been found in the intestinal microbiome of patients with liver cirrhosis,^36^ which could participate in metabolic dysregulated mechanism of NAFLD through the oral-gut-liver axis.^6,11^ Recently, NAFLD was reclassified as metabolic dysfunction-associated steatotic liver disease (MASLD) to emphasize the central role of systemic metabolic dysfunction. Our findings align with this updated framework, as chronic oral inflammation from caries is intrinsically linked to abnormalities that drive MASLD. This reinforces the importance of the oral cavity as a potential intervention target for metabolic liver diseases.^39^

As multifactorial diseases, caries results from interactions between oral bacteria, fermentable carbohydrates, host factors and time, and they may have something common with liver conditions in terms of pathogenesis or have an intermediate mechanism. In terms of the relationship to the oral-gut-liver axis, gut bacteria can have mutual effects with oral bacteria, including cariogenic *S. mutans*, thereby aggravating NAFLD and liver fibrosis through a systematic and hepatic inflammation response together with elevation of disease susceptibility, but the specific pathways remain unclear.^2,41^ Tan et al^46^ recently explored the effect of type 1 diabetes and glycemic traits on increasing the risk of caries, and a combined GWAS meta-analysis dataset of caries was adapted as a replication cohort. Åberg et al^1^ reviewed the bidirectional associations between oral hygiene and liver disorders in 2022, despite focusing primarily on periodontitis. T-cell–mediated immunity downregulation after long-term usage of an immunosuppressive regimen could contribute to the development of caries, but it is specific to the process of transplantation therapy instead of the progress of the liver condition.^14^ Sjögren’s syndrome is an immune-mediated disease common in individuals with chronic hepatobiliary diseases, in which saliva reduction followed by a decrease in salivary proteins and acid buffering capacity could compromise the oral cavity’s natural defense mechanisms against cariogenic bacteria and the retention of fermentable carbohydrates.^16,55^ Patients with active caries were more likely to have xerostomia, lower salivary flow rate, and worse oral and general health conditions.^18^ However, research in 2022 found that primary Sjögren’s syndrome patients had even less frequent liver steatosis and fibrosis.^5^ Unhealthy lifestyles such as high-sugar and high-fat diets and circadian rhythm disruption can lead to metabolic disorders, obesity, NAFLD, and caries at the same time. These results could probably explain why despite positive observational findings being in line with previous studies, our MR analysis did not find statistically significant support for a causal association.

The present study’s advantages are: (1) We combined observational data from NHANES with bidirectional MR analyses; the multi-analytical approach comprehensively evaluates the relationship between caries and liver conditions. This combination allows for the exploration of associations in a large, diverse population and the utilization of genetic data to infer causality, strengthening the robustness of the findings. (2) We extracted data from NHANES 2017–2020, which which contains the most recent data available compared to previous studies. Notably, LSM, the imaging standard to define liver conditions and second only to the gold-standard examination of liver biopsy, was newly collected and applied in our study, providing a more objective and quantitative perspective for assessing liver damage in a large population than typical ultrasound results. (3) We introduced SF and AF into the investigation of caries for the first time, presenting a consecutive view of liver conditions and encouraging further reflection on pathological development.

However, this study also had several limitations: (1) The NHANES study is observational and cross-sectional by design. Therefore, it is impossible to establish strict temporal causality between caries and liver disease. Furthermore, despite comprehensive adjustment for multiple covariates, the potential for residual confounding from unmeasured factors (e.g., detailed dietary composition or genetic predisposition) cannot be entirely ruled out. (2) There is a discrepancy in the definition of caries between our observational and the MR analyses. In NHANES, we exclusively evaluated untreated caries (UDC), whereas the GWAS data for MR utilized the DMFS (decayed, missing, and filled tooth surfaces) index and dentures, representing overall caries experience. This phenotypic inconsistency may partially explain the discrepancy between our statistically significant observational findings and the null MR results, and implies that current MR results reflect the lifetime genetic liability for caries rather than the active inflammatory state of untreated caries. (3) According to the protocol in NHANES 2017-2020, the assessment of caries only includes coronal caries. Hence, data for the more comprehensive caries, such as root caries, is unavailable for analysis. (4) The MR study may have an information bias due to different examinations for individuals with and without NAFLD and fibrosis. (5) NHANES data reflect the situation of US-Americans, while the GWAS and FinnGen data for MR were collected in Europeans; therefore, the discordance of the population in observational and genetic data may lead to biased results and limit our findings to other populations. Further studies using data from the same population could be expected. (6) Finally, we did not apply strict multiple testing corrections (e.g., Bonferroni) to our p-values across subgroup and multi-outcome analyses, which may increase the risk of inflated Type I errors. Our subgroup findings should thus be interpreted as exploratory and hypothesis-generating.

## CONCLUSION

The present observational analysis based on NHANES data indicated that untreated coronal caries was associated with with NAFLD and liver fibrosis. However, MR analysis did not coherently suggest a statistically significant causal link between caries and the increased risk of liver conditions including NAFLD, fibrosis, cirrhosis, and liver fibrosis/cirrhosis, or vice versa. Further cohort studies in a more standardized population should lead to a better understanding of the oral-hepatic interrelationship and underlying mechanisms.

## ACKNOWLEDGEMENTS

This work was supported by the Medical Scientific Research Foundation of Guangdong Province of China (Grant No. A2026121). The authors thank the GLIDE and FinnGen consortium for contributing data as well as all the participants involved in this study. NHANES is conducted by the Centers for Disease Control and Prevention (CDC) and the National Center for Health Statistics (NCHS), and received approval from the NCHS Research Ethics Review Committee. All participants in NHANES provided written informed consent. All genetic information used for this study is publicly available in GWAS summary statistics.

### Appendix

https://www.quintessence-publishing.com/quintessenz/journals/articles/downloads/ohpd_2026_8360_xu_supplements.pdf
